# Biomarkers of cancer angioprevention for clinical studies

**DOI:** 10.3332/ecancer.2015.600

**Published:** 2015-11-24

**Authors:** Adriana Albini, Francesco Bertolini, Barbara Bassani, Antonino Bruno, Cristina Gallo, Stefano Giuseppe Caraffi, Sally Maramotti, Douglas M Noonan

**Affiliations:** 1 Laboratory of Translational Oncology, Department of Research and Statistics, IRCCS Arcispedale Santa Maria Nuova, Reggio Emilia 42123, Italy; 2 Laboratory of Haematology-Oncology, European Institute of Oncology, Milan 20141, Italy; 3 Scientific and Technology Park, IRCCS MultiMedica, Milan 20138, Italy; 4 Department of Biotechnology and Life Sciences, University of Insubria, Varese 21100, Italy; * These authors share equal contribution

**Keywords:** angiogenesis, chemoprevention, therapy, inflammation, circulating endothelial cells

## Abstract

With the great advances made in the treatment and prevention of infectious diseases over the last century, chronic degenerative diseases—cardiovascular, cerebrovascular, and cancer—represent the major causes of death in the developed world. Although massive efforts and investments have been made in cancer therapy, the progress made towards reducing mortality has been more successful for cardiovascular disease than for tumours. This can be attributable largely to an active prevention approach implemented for cardiovascular disease. Cardiologists treat their patients before the overt disease becomes life threatening, performing early interventions in phenotypically healthy patients, by using several markers that predict risk. If the concept of prevention could be applied to cancer in a more extensive way, a significant number of tumours could be avoided through preventive measures. Prevention approaches range from avoiding tobacco exposure to dietary strategies to active pharmacological approaches in higher risk groups. Host targets rather than the tumour cells themselves are attractive for chemoprevention, in particular endothelial and immune cells. Angioprevention i.e. preventing cancer angiogenesis is a key concept that we introduced; yet one of the major current challenges for anti-angiogenesis in therapy and prevention is finding the right biomarkers. Here we discuss the importance of angioprevention and the potential use of VEGF, PlGF, CD31, Ang and Tie, circulating vascular cell precursors, and microRNA as potential biomarkers.

## Introduction

The concept of pharmacological cancer prevention, known as chemoprevention or preventive therapy, is not new [1] and should be of important benefit for the public [2]. However, progress in this area has been hampered by several problems. Many of the original studies target the cancer cells, blocking DNA damage and mutagenesis [3]. However, prevention in a healthy individual, particularly given the diversity of cancer risk factors and the variability with which they are associated with tumour insurgence, means that the host itself might represent a better target than the tumour. Recently, our thinking in oncology has evolved from a simplistic view from where tumours are just a hyperproliferation of mutated cells to a more holistic concept of cancer as a tissue. This being composed of initial cancer ‘seeds’ and stromal components, matrix molecules, and numerous host cells that make up the tumour mass. Effective prevention should start when a tumour is not yet detectable or even before it forms, and therefore could be aimed at the microenvironment, using intervention strategies that must have little or no side effects.

### Angiogenesis

The proliferating tumour cells need host support, including elaborating a vasculature for tumour expansion, a stromal scaffold, and an inflammatory ‘polarised’ infiltrate in a situation of constant tissue reconstruction [4–11]. These host components are considered targets for therapy and prevention, and these are particularly appealing in that they are normal, untransformed cells, allegedly less prone to develop resistance to therapeutics. Efforts in finding prevention targets have been aimed to the main process by which tumours form a new vasculature permitting their growth: angiogenesis. Pietro Gullino, Judah Folkman, Harold Dvorak, Napoleone Ferrara, Doug Hanahan, and other giants in the field hypothesised that blocking the expanding tumour vasculature would hamper tumour growth by essentially starving the tumour cells themselves. This area led to extensive basic research and about 30 years later to clinically approved anti-angiogenic drugs [4–6].

### Angioprevention

We observed that most compounds with known or putative cancer chemopreventive activity also show significant anti-angiogenic activities, a concept we termed angioprevention [12]. The anti-angiogenic properties of these compounds is likely a common and critical effect, responsible for a substantial portion of their chemopreventive activity [12–14]. Closely linked to this is inflammation, as many of these chemopreventive compounds inhibit both angiogenesis and inflammation [10, 15], apparently by frequently targeting common molecular pathways (see below). We also defined four levels of angioprevention [13]. Extensive epidemiological studies have recently indicated that the biguanide metformin, currently the first-line therapy for type 2 diabetic patients worldwide, can reduce the incidence and severity of several types of cancer [16–27], while other anti-diabetic drugs had no effect on cancer incidence. We have recently reported that metformin can reduce angiogenesis *in vitro* and *in vivo*, and it is particularly efficient in the context of obesity [28]. Further, we have shown that metformin targets *in vitro* and *in vivo* both breast cancer (BC) cells and white adipose tissue (WAT) endothelial progenitor cells, as well as WAT progenitor cells [28, 29]. We found that another biguanide phenformin was significantly more active in inhibiting angiogenesis than metformin, both *in vitro* and *in vivo* [29]. However, phenformin was associated with rare, but sometimes fatal acidosis that caused its withdrawal from clinical use in diabetes [30, 31].

### The immune system and inflammation

Immunity is now a recognised target for cancer therapy with the great success of drugs directed against the immune check point blockade such as CTL4, PD1, and PD-L1 [32]. Inflammation has also become a leading field, in particular owing to the notion that innate immunity cells can be reverted from a pro-angiogenic, pro-tumour polarisation to an anti-angiogenic, anti-cancer phenotype [8, 9]. This concept is even more relevant in cancer prevention, since inflammation can be considered a ‘promoting’ event in carcinogenesis [8–11, 13, 15, 33, 34]. Recent data indicate that targeting host tissues and in particular the cancer microenvironment [10, 13] will be the key for successful cancer chemoprevention.

### Prevention within the tumour microenvironment

There has been a rapid expansion of studies focusing on the tumour microenvironment, which is a complex society of many cell types [10]. These include endothelial cells and endothelial cell precursors, pericytes, smooth muscle cells, fibroblasts of various phenotypes, innate immune cells (largely macrophages, neutrophils, mast cells, and dendritic cells) and even specific immune cells (T, B, and natural killer (NK) lymphocytes) [4–11, 35]. All of these cells have been suggested to participate in tumour progression. Thus, the carcinogenesis process can be regarded as a series of events involving an entire tissue or organ. Control of the reactive microenvironment within a developing tumour is as important as the knowledge and control of the dysfunctional tumour cells themselves. Several anti-inflammatory agents have been linked with cancer prevention [10, 13], suggesting that inflammation is a common factor.

### Targets of angiopreventive compounds

Phytochemicals and diet-derived drugs are under increasing investigation for their potential in cancer prevention. Plant-derived compounds frequently target host cells at diverse molecular hubs that permit these molecules to have broad, efficacious effects with little or no toxicity. Chemoprevention can be targeted to the microenvironment by using phytochemicals that display anti-angiogenic and anti-inflammatory activities in an angiopreventive approach. Metformin, which has been epidemiologically associated with reduced incidence of several cancers and with angiogenesis inhibition (see above), is a synthetic molecule based on the observation that goat’s rue/French lilac (*Galega officinalis*) had anti-glycemic effects.

Immune cells are also compelling targets for angiogenesis inhibition as they often ‘orchestrate’ angiogenesis on account of whether they are polarised to a pro- or anti-angiogenic state [35]. Rapidly expanding data from many laboratories, including ours, place immune cells at the centre of endogenous angiogenesis regulation, highlighting their role as a pharmacological target also in prevention [35]. In keeping with this, one of the most widely used pharmaceuticals and a known cancer chemoprevention compound is a plant-derived drug: aspirin [13], a modification of salicylate from willow bark.

**Figure 1.**
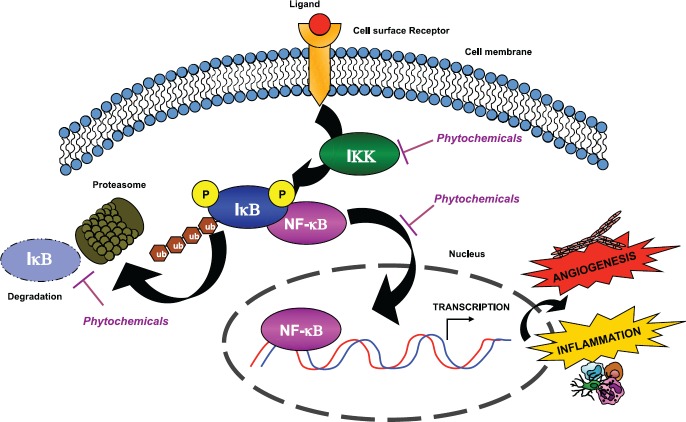
Phytochemicals inhibit the NF-κB pathway. Chemopreventive compounds are able to suppress NF-κB activity by inhibiting its nuclear translocation. NF-κB is normally associated with its inhibitor IκB. The activation of IKK leads to IκB phosphorylation and subsequent degradation by the proteasome. Thus, NF-κB is released and translocates to the nucleus, where it is able to bind consensus sequences that lead to the transcription of pro-survival, inflammatory, and angiogenic molecules. Widely used phytochemicals are able to interfere with NF-κB activation, mostly by targeting IKK activity and decreasing the phosphorylation of IκB.

A common pathway targeted by many phytochemicals is the NF-kB pathway [10, 13], a master regulator of both inflammation and angiogenesis (Figure 1). Another pathway targeted by some phytochemicals involves serine/threonine kinase Akt signalling, which is intertwined with the NF-kB pathway.

The signal transducer and activator of transcription (STAT) family consists of seven members—STAT1, STAT2, STAT3, STAT4, STAT5A, STAT5B, and STAT6—encoded by distinct genes. These proteins were discovered as molecules mediating cytokine signalling: receptor activation of a STAT protein has a very strong influence on immune cell polarisation within a specific microenvironment. Recently, STAT3 has been indicated as a key element in driving the immunosuppressive, pro-angiogenic, pro-tissue reconstruction, and pro-survival microenvironment that is characteristic of many tumours [36]. Inhibition of STAT3, like repression of NF-kB, may be a common pathway involved in the tumour suppressive action of many phytochemicals. This implies that these molecules act by targeting not only the tumour cells, but also the tumour microenvironment.

Another fascinating mechanism shared by many phytochemicals is related to their effects on cellular metabolism. Molecular targets of phytochemicals include activation of sirtuin 1 (Sirt1), mTOR and glycogen synthase kinase 3 (GSK-3β), which are important regulators for both metabolism and cell survival [14]. Since most tumour cells depend on glycolysis even in normoxia, a concept known as the Warburg effect, additional alterations in energy balance can further destablise tumour cells and enhance cell death [14].

## Biomarkers

Biomarkers are essential tools to monitor disease progression and disease treatment. In oncology, biomarkers can be used for diagnosis, prognosis, treatment selection, monitoring, and follow-up. They are particularly important in targeted therapy, where evaluation of tumour burden is poorly indicative of response. Biomarkers would be particularly useful for cancer prevention, permitting clinicians to monitor the presence of risk-related molecules, and their modulation by preventive agents. As we shall see, however, there is currently a paucity of potential biomarkers for prevention and angiogenesis.

### Biomarkers of angiogenesis

Several anti-angiogenic drugs, including monoclonal antibodies and TKI inhibitors, have been approved over the past ten years for the therapy of a variety of different types of cancer. These include advanced colorectal, breast, lung, kidney, central nervous system, ovarian, cervical, neuroendocrine, and thyroid cancers [4, 37–39]. Most of the biological and clinical activities of the currently available generation of anti-angiogenic drugs target vascular endothelial growth factor (VEGF) and its related pathways. However, the clinical benefits associated with these anti-angiogenic drugs are limited to a few months, and a large majority of treated patients have subsequently suffered disease progression [37]. It is still unclear whether the slight impact on patient’s overall survival (OS) and/or progression-free survival (PFS) is caused by a small clinical benefit in every patient or a larger benefit in just a small subpopulation of patients.

**Figure 2.**
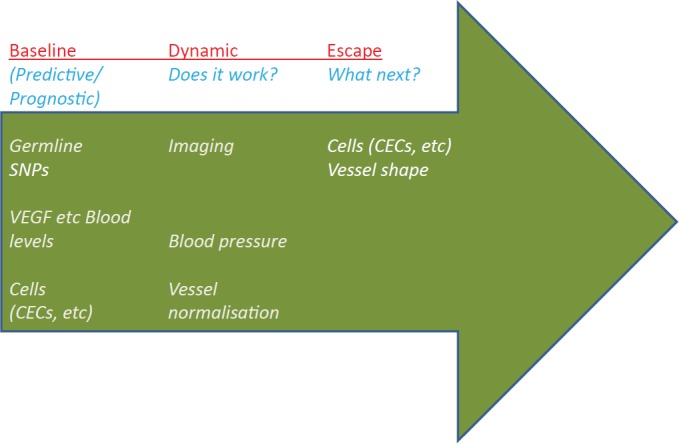
The three main categories of angiogenesis-related biomarkers that are currently under clinical validation. Predictive/prognostic biomarkers include a) blood levels of angiogenesis-related growth factors and receptors (e.g. VEGF and VEGFR, FGF and FGFR, Angiopoietins and related receptors); b) germline SNPs evaluation of the molecules mentioned above; c) circulating cells (CECs and CEPs). Currently investigated dynamic biomarkers, potentially useful to ascertain whether a given therapy is actually offering a clinical benefit to the patient, include blood pressure, evidence of vessel pruning (or, vice-versa, normalisation), perfusion-related parameters acquired by MRI. Finally, an emerging new class of potentially useful biomarkers includes those suggesting an initial escape from the ongoing therapy, and thus the need to change therapy. CEC/CEPs and immunohistochemistry (IHC) evaluation of the generation of dysplastic ‘supervessels’ are currently investigated pre-clinically and clinically with this goal in mind. All these biomarkers are covered in references [4, 37–39, 50].

Biomarkers for anti-angiogenic therapy are needed in order to select patients likely to respond and avoid those likely to do worse, as well as to optimise therapy dose/schedule, and to monitor therapy and determine when escape is likely to occur. Numerous trials have included many different kinds of biomarkers, from proteins to physiological ones (for example hypertension), however there is no consensus for angiogenesis biomarkers (Figure 2). Blood levels of VEGF have been investigated in numerous studies, along with related molecules such as placenta growth factor (PlGF). Several papers have found a lack of correlation between VEGF levels and response to anti-angiogenic therapy, while others noted an increase in VEGF and PlGF levels with therapy [4, 38, 39]. Although circulating endothelial cells (CECs) seem to be a promising marker, development has been hindered by the overlap in antigens between CECs, platelets, and some haematopoietic cells. Identification of other cells, such as circulating endothelial progenitors (CEPs), is also controversial [38].

Recently, a study suggested that Angiopoietin1 (Ang1) and Tie2 levels could predict the response to the VEGF-inhibitor bevacizumab (Avastin)—in combination with standard chemotherapy—in patients with ovarian cancer [40]. This study investigated numerous angiogenesis markers and found that patients with high Ang1 and low Tie2 responded well to bevacizumab, those with high Ang1 and high Tie2 did worse on bevacizumab than those without, while low levels of Ang1 did not influence outcome [40]. It remains to be determined whether this can predict response and if this can be applied to different cancers treated with bevacizumab or other anti-angiogenic agents.

In a study on a large number patients with advanced pancreatic cancer treated with bevacizumab plus chemotherapy, proteomic analysis of the plasma suggested that low levels of Ang2 and stromal-derived factor 1 (SDF1) are potential predictors of primary resistance to bevacizumab [41]. The applicability of these biomarkers to different antiangiogenic drugs or cancer types is controversial, though in patients with advanced renal cell carcinoma (RCC), low plasma levels of Ang2 were found instead to indicate a better response to tyrosine-kinase inhibitor sunitinib used in combination with chemotherapy [42].

The lack of biomarkers suitable for selecting the patients who are most likely to benefit from these targeted therapies is currently impeding a more rational use for this class of drugs in adequately selected patient populations where a clinical benefit might be predicted. Moreover, the same lack of validated biomarkers severely limits the ability to determine optimal biologic dose and schedule for these drugs.

### Searching for new biomarkers

**Figure 3.**
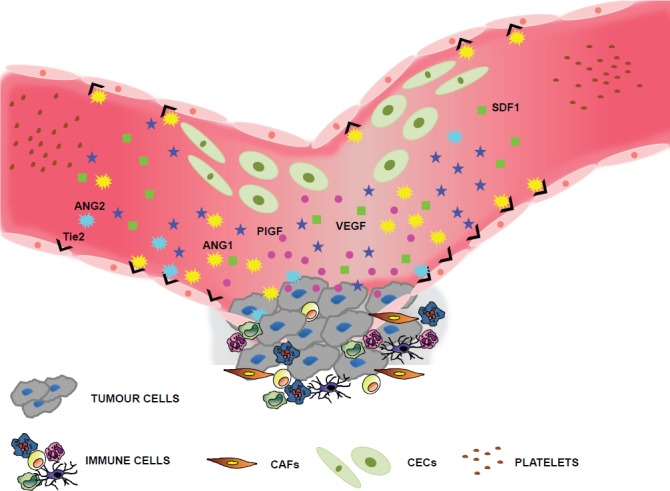
Selected biomarkers of angiogenesis. Many laboratories look for biomarkers that can be traced in the peripheral blood, since they would be relatively non-invasive and convenient for keeping track of patient responses to antiangiogenic treatments. These biomarkers include soluble factors secreted by both tumour and stromal cells, such as VEGF or PlGF, or microenvironment-derived proteins such as SDF1 or the Ang1/Ang2/Tie2 ligand-receptor system. Novel, promising strategies involve the detection of circulating cells such as CECs or CEPs, although specific surface antigens for their unequivocal discrimination still requires validation [37, 38].

Numerous laboratories across the globe are now looking into other types of markers for response to therapy, in particular anti-angiogenic therapy, which will also shed light on their angiopreventive effects. Candidate predictive biomarkers include germline single nucleotide polymorphisms (SNPs) of angiogenesis-related growth factors and receptors such as VEGF, VEGFR, PlGF [43, 44], and expression of microRNAs and other non-coding RNAs (Figure 3). Interestingly, recent studies suggest that several phytocompounds are able to modulate the expression of microRNAs [45–47]. The effects that phytochemicals exert through miR modulation are likely to contribute to the overall performance of these molecules.

A retrospective analysis of the phase III GOG-0218 trial has identified CD31 expression as a potential biomarker associated with improvements in PFS and OS for patients with advanced ovarian cancer treated with bevacizumab plus chemotherapy [48].

Many imaging techniques are currently under clinical or pre-clinical evaluation as a non-invasive approach to provide surrogate markers of angiogenesis or response to anti-angiogenic treatment. They include dynamic contrast-enhanced magnetic resonance imaging (DCE-MRI) to assess vessel perfusion, permeability, and density; diffusion-weighted MRI (DW-MRI) to monitor tissue cellularity, integrity, and extracellular space complexity; DCE-ultrasound (DCE-US) imaging using microbubbles targeted to VEGFR or vascular integrins to measure microvessel density and blood flow/volume; positron emission tomography (PET) to image hypoxia as a key driver of angiogenesis; and novel techniques such as vessel architectural imaging (VAI) to estimate vessel caliber [49]. An in-depth discussion of these methods is beyond the scope of this paper and can be found in specialised reviews [50–53].

## Conclusions

The identification and validation of biomarkers for angiogenesis and angioprevention are urgently needed for clinical applications. In this context, some promising avenues are derived from an increasing knowledge of the variability of humans, largely achieved through non-biased ‘omics’ search approaches. In the near future, these should significantly contribute to identifying what patient types are the most likely to benefit from an angiopreventive approach.

## Contributions

**Adriana Albini and Francesco Bertolini** authors share equal contribution.

## Conflict of interest

The authors declare no conflict of interest.
